# Deconstructing institutional racism and the social construction of *whiteness*: A strategy for professional competence training in culture and migration mental health

**DOI:** 10.1177/13634615221087101

**Published:** 2022-04-04

**Authors:** Felicia Lazaridou, Suman Fernando

**Affiliations:** 1 Department of Psychiatry and Psychotherapy, 14903 Charité University of Medicine Berlin, Berlin, Germany; 2 School of Social Sciences, 4904 London Metropolitan University, London, UK

**Keywords:** decoloniality, institutional racism, intersectionality, mental health, *whiteness*

## Abstract

The position presented in this article draws on the professional insights of the authors, reflecting on issues of global political importance in culture and migration mental health. As institutional theory perspectives continue to develop, solutions to complex social problems such as racism require embodied knowledge if the lines of authority and basic occupational routines are to be meaningfully renegotiated. Embodied knowledge is socially situated and self-reflexive and reflects cumulative and marginalized experiences that contribute to a better understanding of institutional racism and the social construction of *whiteness*. The authors foreground the development of critical consciousness and emotional literacy in order to be more professionally competent in institutional contexts of mental health training, education and practice. To this end, elements of due process, transparency, inclusiveness, community engagement and accountability are at the center of a political and intellectual movement towards epistemological justice to promote antiracism and social justice in culture and migration mental health. The authors define *decolonial intersectionality* as a clear philosophical vision outlining how best to respond to those at risk of experiencing racism and the associated mental health burdens.

## Introduction

Racism, when it is embedded in the structures, policies and practices of our social and political institutions can be termed “institutional”. *Institutional racism*, which will be described by the authors more fully below, is reflected in professional practice and working methods that result in racialized disparities. Frantz Fanon, psychiatrist and political philosopher, stands out as one of the earliest academics to explore the nature of racism from a psychosocial perspective. Fanon (1967) talked of “vulgar racism in its biological form”, which was evident for several hundreds of years, being replaced in the mid-20th century by “more subtle forms” (p. 35). In a study of Fanon's clinical psychology and social theories, McCulloch (1983) refers to this new racism as “cultural racism”—describing this as “a more sophisticated form [of racism] in which the object is no longer the physiology of the individual but the cultural style of a people” (p. 120). Cultural racism believes that the dominant group's culture is superior to the seemingly “lower” minority groups.

Cultural racism champions the supremacy of cultures. Commonly, some version of European culture or, more specifically, *white* European culture, rather than the *white* “race” (Amin, 1989), thereby producing a situation of racism without “races” (Balibar, 1991). It was the American civil leaders Stokely Carmichael (later Kwame Ture) and Charles V. Hamilton in their 1967 book, *Black Power: The Politics of Liberation* (Carmichael & Hamilton, 1967), who first described institutional racism:

It takes two, closely related forms … we call these individual racism and institutional racism. The first consists of overt acts by individuals … the second type is less overt, far more subtle, less identifiable in terms of specific individuals committing acts … and thus receives far less public condemnation than the first type. (p. 2)

Institutional racism forms an array of broader structural racism processes “that exclude … substantial numbers of members of particular groups from significant participation in major social institutions” (Henry & Tator, 2005, p. 352). According to minority mental health models like the racism-induced reactive negative emotionality cycle (Lazaridou & Heinz, 2021), structural racism and institutional racism result in experiences of rejection and emotional alienation in public spaces for Black people and People of Color (Lentin, 2015).

Structural racism in employment, earnings and credit may mutually limit equal access to quality, affordable accommodation. However, when public spaces are sites of surveillance, intimidation and frequent hostility by the police or by ordinary citizens, then the structure of social situations, such as even leaving one's house and speaking in public, are filled with stress, anxiety, and fear (Chou et al., 2012; Sibrava et al., 2013). There is pervasive evidence that structural racism has destructive impacts on the health and wellbeing of patients from minority groups, including migrants, refugees, and asylum seekers (Alvarez et al., 2016; Bailey et al., 2017; Graham et al., 2016; Noh & Kaspar, 2003). Racism can also become supported and reinforced by the exclusion of minority groups from deliberations over the needs and priorities of the population in planning and designing public spaces; which result in disparities relevant for living conditions, including inadequate sanitation facilities, exposure to pollution and a lack of green, open spaces—amenities that are influential for health and well-being (Rishbeth et al., 2017).

Paradoxically, processes of claiming racialized identities can occupy a wide range of moral responses to institutional racism or serve the broader purpose of challenging derogatory labels (Appiah, 1996; Fanon, 1952/2008). The multiple forms of racism's tacit and explicit “knowing” regarding prejudicial attitudes based on the idea of “race” and “races” are acquired through socialization, externalization, combination, and internalization (Bonilla-Silva, 1994; Omi & Winant, 2015). The naturalization of this epistemic injustice is one detrimental effect of experiencing racism in domestic, communal, professional, and international contexts (Oppong, 2019). Learned helplessness (Seligman, 1975, 1978), which in the context of racism, is the incorporation of denigrating theories concerning innate intellectual inferiority (Jensen, 1969) and inferior personality structure (Kardiner & Ovesey, 1962) in one's worldview, has the potential to produce profoundly adverse effects on mental health and well-being (Williams & Mohammed, 2009). The costs of racism to children, youth, adults, and older adults are well documented (Greer & Spalding, 2017; Jones & Neblett, 2016; Pachter & Coll, 2009). Given substantial empirical evidence, scholars have conceptualized racism as a social determinant of mental health (Medlock et al., 2017).

## Racism and mental health symptoms

As a result of deep regret and remorse following the Jewish Holocaust and anti-racism movements worldwide, a significant ideological shift in “race”-thinking occurred from the 1950s onwards: from biological determinism to social constructivism (Brattain, 2007; UNESCO, 1950; United Nations, 1965). “Race”-based perceptions gradually shifted from focusing on the heritability of genetics underpinning intelligence and personality to social contexts’ environmentalism (Sternberg et al., 2005). This ideological shift is associated with legislation and public policy-level responses to racism-motivated crimes only to a limited extent (Nwabuzo, 2019). According to data from the European Network Against Racism (2014–2018), rates of reported racism-motivated crimes are increasing in many of the 24 EU member states included in the report; anti-Semitism being of particular concern in France, the UK, and Germany while Antiziganism is a specific concern in the Czech Republic, Bulgaria, and Slovenia (Nwabuzo, 2019).

Social and political institutions and organizations across Western societies have been coming under increasing pressure in recent years to hold themselves infra-structurally and reflexively accountable for their lack of sufficient responses to the racism that causes civil society organizations to fill in the gaps. Even more so since the rise of the Black Lives Matter (BLM) movement after the murder of George Floyd in the United States (e.g., see The Society for Adolescent Health and Medicine, 2018). Consequently, research suggests that civil society organization workers are susceptible to burnout due to the pressure of working in under-funded and under-resourced environments (Ziersch & Baum, 2004). In small to moderate duration and amounts, stress can facilitate a drive to succeed; however, burnout is a state of emotional exhaustion caused by stress overload (severe duration and amounts) (Lazaridou, 2021). It may be unfair to place the burden of filling gaps in civil society on health organizations, just as it may be unrealistic to suggest that professionalized Western mental health care services are capable of providing a healing space for injustices in which they have played a key role historically (see Heinz, 1998, for a discussion of the coloniality of knowledge in psychiatry). In any case, if civil society organizations are to be a part of the solution, they will need more resources (Kelly, 2021).

A recent systematic review and meta-analysis suggests burnout leads to stress-associated affective disorders such as depression, *r* = .52, *SE* = 0.012, 95% CI  [0.492, 0.547] (Koutsimani et al., 2019); the Pearson's correlation of *r* = .52 is a medium effect size (Cohen, 1988). The need for action has become all the more urgent in recent years. In the context of the Black Lives Matter movement, researchers have been investigating how the political climate harms the mental health of Black people and People of Color via a stress proliferation paradigm (Berger & Sarnyai, 2014; Wallace et al., 2016; Williams, 2018). Researchers have discovered adverse mental health outcomes in the general Black population in the first and second months following the death of each unarmed Black man by the police (Bor et al., 2018). To clarify, a study of 302 African American and Latinx 11–19-year-old adolescents investigated associations between exposure to traumatic events online and depressive symptoms and post-traumatic stress (PTSD) symptoms (Tynes et al., 2019).

Tynes et al. (2019) conceptualized traumatic online events as (1) exposure to images or videos of someone from one's own racialized group getting beaten during the past year, (2) exposure to images or videos of someone from one's own racialized group getting arrested or detained during the past year, and (3) exposure to viral videos of a Black person getting shot by a police officer. With respect to the main effects, exposure to traumatic events online was more strongly associated with PTSD symptoms (*b* = .28, *p* < .01) than to depressive symptoms (*b* = .12, *p* < .05). The bivariate correlations indicate a small effect size between racism and mental health. This research finding confirms a meta-analysis by Paradies et al. (2015), who found overall that racism is associated with mental health symptoms, *r* = −.23, 95% CI [−.24, −.21], *k* = 227). Small effect sizes in this context highlight the importance of understanding minority mental health by considering the influence of ideology-related information culturally transmitted between and within racialized groups and communities (Fernando & Keating, 1995). Substantive similarities that serve as the foundation for positive racialized identity development, such as the value of shared experiences, including shared states of mind and emotions, may help mitigate the negative effects of racism (Loyd et al., 2021).

## Accountability—A process of transformative and social justice leadership

Decolonizing academia requires the greater inclusion of knowledge produced by Black people and People of Color and increasing their representation in positions of power within academia (Rodríguez, 2018). Deconstructing institutional racism at the core of the academy is an integral part of decolonization (Bell et al., 2021). *Whiteness* has been fundamental to the non-performativity of “anti-racism” work within the pre-existing structures of action planning and resources allocation at the highest levels (senior administration and governance) (Ahmed, 2006). Consequently, despite the continued revolutionary migration of antiracism scholarship from the margins of policy and politics into the mainstream, the *whiteness* of such “anti-racist” work ought to be scrutinized as potentially damaging and unequal for Black people and People of Color (Doharty et al., 2021).

By focusing on spaces of education for psychiatrists, clinical psychologists and allied mental health professionals, in this article we consider the highly problematic naturalization of *whiteness* in Western academic institutions that results in faculty representations and curricula reflecting the absence of non-*white* minority bodies, minds, and discourses. Racialized categories themselves perpetuate monolithic perspectives about the social heritage of diverse groups of people by assuming that each shares a particular country of origin, skin color or legacies of collective trauma (Fassin, 2011). However, though the meanings ascribed by racialized categorization have been described as “factish” by some (Erasmus, 2019, p. 495), they are a contingency. In philosophy and logic, racialized categories are a contingency that illustrates that they are neither verifiable nor refutable (Heinz et al., 2014). Nor are they fixed from one context to another (Fassin, 2011).

Racialized people are free to make their own choices regarding belongingness, and the purposes and consequences of that (Sen, 2006). Transformative and social justice leadership can empower communities of struggle to find their meanings by focusing on individuals and morally grounded relationships (Foster, 1989). We suggest that successful accountability is more than just holding the leaders of respective Western institutions to account for the non-performativity of speech acts they express in the name of their institutions (Ahmed, 2006).

We argue that successful accountability is about active institutional reorganization, thereby dismantling existing power structures of *whiteness* and installing a culture cultivating intercultural teamwork, exchange, and mediation across hierarchical relations (Bilge, 2013). In place of empty promises, social justice leadership would promote equity and place more excellent value on inclusive processes for effective anti-racism policy development and implementation to advance equal opportunities with respect to participation and recognition (Boykin et al., 2020). To prevent asymmetry from re-emerging, transformative and social justice leaders need to commit fully to the values of a decolonial agenda (Foster, 1989), which encompasses the critique of self and social conditions to continuously promote possibilities of genuine inclusivity (Shields, 2004). This is due to the colonial “social imaginary significations” on which and within which Western mental health science was founded, which subjugate racialized people (Bulhan, 2015; Kilomba, 2008; Mignolo, 2000; Oppong, 2019). When comparing the characteristics of economic imperialism to political or intellectual imperialism, it becomes clear how the apparatus for defining the quasi-pathological status of the racialized people was hegemonically constructed in order to valorize activity focused on maintaining the colonizer's economic dominance (Mosse, 1985). As Tzvetan Todorov argued in the preface of Edward Said's *Orientalism*, ever-evolving racialized hierarchies within systems of subjugation are a position of dominance when they speak *for* the colonized, that is to say: “‘I have the truth about you’, it is informing the nature of my knowledge, but it is also a relationship in which ‘I’ dominate and the other is dominated” (Said, 1978, p. 3, cited in Maitre, 2011). This is what the canonization of ethnocentrism achieves, according to Mbembe (2015): “The knowing subject is thus able, we are told, to know the world without being part of that world and he or she is by all accounts able to produce knowledge that is supposed to be universal and independent of context” (pp. 9–10; Oppong, 2019).

The implication is that, regardless of who is espousing Western mental health science, whether they are good-willed members of the dominant native Core, *white* Leftists, decolonialists, or people excluded at the Periphery, without reflecting on and incorporating the indigenous traditionalism of local communities, Western mental health science’s global expansion's positionality as political imperialism remains intact (Ratele et al., 2018). That said, forms of political or intellectual coloniality in academia are perpetuated by a lack of embodied representation both inside and outside of migrant and native situatedness (Arghavan et al., 2019).

## The cultivation of internalized racism and self-stigma

A large body of literature, including a large meta-synthesis of quantitative and qualitative research analyzing 144 studies and 90,189 participants, is available on the nature of mental illness stigma (Clement et al., 2015). Summarizing the social concept of causal beliefs and the momentum of Goffman's work, community-based research on stigma has intrinsic value and is of strategic importance (Goffman, 1963; Tawiah et al., 2015; Von Lersner et al., 2019).

According to Evans-Lacko et al. (2011), how people become influenced to think about their mental illness shapes how they evaluate and perceive their own nature (Baumeister & Bushman, 2009). In society and community, fear and stigmatization of the mentally ill developed based on negative societal schemas, which were constructed, for the most part, through derogatory depictions in public media (Thornicroft, 2006).

Cultivation Theory is an influential values orientated approach to understanding the impact of mass media communication on people's perceptions and expectations (Gerbner, 1998), and has been used to explain public media's contribution to mental illness stigma (Gerbner et al., 2019). Herman and Chomsky (1988) advanced an understanding of how public media became a technique to manipulate all persons. Derogatory comments, for example, linking the majority of mass shootings carried out by *white* men in America to mental illness exacerbate public mental health stigma for the mentally ill. A recent study found that perpetrators’ skin color influenced the portrayals of the criminal in public media. Empathy was extended to *white* shooters described as “mentally ill” compared to Black shooters who were framed in the context of their so-called “dangerous Personality” (Duxbury et al., 2018). Perhaps of even greater concern, a recent study by the U.S. Federal Bureau of Investigation in the context of complex social dynamics questioned the link between mass shootings and mental illness, describing the link as false and socially disadvantageous to an already stigmatized population (Silver et al., 2018). According to cultivation theory, as various social justice movements gained traction, the elites’ ability to impose social control and garner “consent” from the oppressed by enacting inequitable government policies and using military force became less available, necessitating the cultivation of control through public stigma (Herman & Chomsky, 1988).

Although institutional racism within mental health services is not a universally experienced barrier to mental health care for Black people and People of Color, due to the globalization of public media, the internalization of racist connotations at the intersection of skin color and mental health stigma is an increasing phenomenon across the Global North and South (Gyamfi et al., 2018). This also affects migrant populations, as seen, for example, among Black people and People of Color in the German mental healthcare systems and German hospitals. They experience the additional psychological stress of oppression and chronic racism alongside the consequences of the stigma of mental illness disseminated through public media, contributing to their social ostracism, economic disadvantage, and impaired physical health (Berzins et al., 2003, Sharac et al., 2010; Stuart, 2006). More empirical research is needed to inform training in culture and migration mental health (Schouler-Ocak & Aichberger, 2017; Schouler-Ocak et al., 2019).

## The social construction of *whiteness*

The emerging academic field of critical *whiteness* studies and the gradually increasing uptake of critical *whiteness*-based training in professional environments is a testament to a reflexive turn toward accountability in more liberal parts of super-diverse societies (Foste, 2020). Critical *whiteness* training sessions are rooted in the critical race scholarship field to improve one's performance in consciously recognizing and addressing structural and institutional level issues of *whiteness*, power, and privilege (Nayak, 2007). Given the predominance of *white* thinkers in the development of theories and paradigms that underpin Western psychiatry and clinical psychology, any exploration of institutional racism in how psychiatrists and clinical psychologists develop their diagnostic, therapeutic, and research skills requires the critical analysis of *whiteness* within mental health (Wood & Patel, 2017).

The positive psychology paradigm, for example, is typically credited to *white* psychologists, including Peterson and Seligman (2004) and Csikszentmihalyi (2014). However, because of wanting to conceptualize and elaborate on the deleterious effects of racism, Black scholars have, over the years, made significant contributions to the strengths-based approach to mental health (Caldwell-Colbert et al., 2009). The strengths-based model in minority mental health focuses on recovery, emphasizing the value of balance, reflection, energy, association, health, and empowerment (e.g., Evans et al., 2017). Nevertheless, stakeholders at all levels throughout the academy have automatically discounted numerous Black psychologists’ contributions by (re)enacting prejudices about genetic differences in intelligence**.** Learning to recognize *whiteness* as “a constantly shifting location upon complex maps of social, economic and political power” (Ellsworth, 1997, p. 264), alongside the necessary cognitive and emotional transformations all the while guided by intersectional reflexivity, enables successful accountability and is part and parcel of institutional reforms that result in equity in Western mental health systems (Baima & Sude, 2020).

## Intersectionality

The emotional strain of a marginal existence at the juncture between the democratic liberalism and the collective racism of *white* feminism was the catalyst for intersectionality perspectives woven throughout the history of Black feminist discourse (see Sojourner Truth's 1851 speech *Ain't I A Woman?* referenced by hooks [1981]). Black feminist scholars focus on the social construction of racism and form critiques that draw inspiration from the conceptual toolbox of Kimberlé Crenshaw's (1989, 1991) intersectionality theory to counter the hegemonic narratives told by the political subtext of institutionalized *whiteness*. Another focus of Black feminist scholarship continues to explore how the interconnectedness of the dominant narratives across institutions in society forms a “public transcript” (Scott, 1990, p. 45), laden with denigrating theories that reinforce binary thinking through which the exclusion of Others becomes defended (May, 2015). This “public transcript” represents the discursive constructions of Black people and People of Color embedded in the subtext of broader society, as mediated through the trialectics of knowledge, space and identity, both historically and in the present (Henry & Tator, 2005; Song, 2014).

The interdisciplinarity of Black feminism discourse has strengthened the analysis of multiple forms of discrimination on mental health, experienced simultaneously and separately in people's daily lives (Adames et al., 2018; Taylor & Richards, 2019). The feminist perspective of the 1990s raised awareness of just how easy it is for those in privileged positions—at the top of real and imagined social hierarchies—to ignore the knowledge, experiences, and testimonies of those situated differently in structurally disadvantaged positions (Harding, 1997; Hekman, 1997; Collins, 1997; Smith, 1997). Intersectionality arose as a framework and method of inquiry in feminist literature. It was interested in how people are situated unevenly within different, converging social identities; and how those intersections (re)produce social inequalities (Crenshaw, 1991). In situating intersectionality as a tool of social justice capable of profoundly enabling institutions and organizations to take accountability, Bilge (2013) makes the important point that *whiteness* harms society by perpetuating the assumed authority of the more structurally advantaged thinkers and their knowledge.

Bilge (2013) draws on the concept of the “*white* habitus” (p. 412) to outline how the *whiteness* of knowledge and power detrimentally obscures any and all understandings about the situatedness of Black people and People of Color. This situatedness means that it is more likely for Black people and People of Color to be aware of certain things and pose the crucial questions than it is for *white* people (Bonilla-Silva et al., 2006). As applied in describing the *whitening* of intersectionality, the phenomenology of *whiteness* encompasses the socialization processes of *white* people around whom Western opportunity structures and public institutions are oriented (Ahmed, 2007; Bilge, 2013; Bonilla-Silva et al., 2006). The notion of *white* privilege is the privilege of not being the target of structural or institutional racism (McIntosh, 1988). In this context, Bilge (2013) asserts that intersectionality's transformative potential is often blocked by the hostile and resistant patterns of behavior of those occupying the privileged normative standpoint of *whiteness*.

A model by Watt (2007) proposes that a privileged standpoint encapsulates a social position that extends beyond the actualities of daily experiences; it articulates that it is worthwhile to consider privilege as a standpoint that frames the way structurally advantaged people perceive themselves and others (Leonardo, 2002, 2004). The theoretical perspective that Bilge (2013, p. 412) terms “the *whitening* of intersectionality” describes a process in which *white* people who are resistant to intersectional reflexivity exhibit various defense modes and co-opt a theory originating from the lives of Black women for their own neoliberal “but enough about you, let me tell you about me” purposes (Apple, 1998, p. xi; Lewis, 2009; and May, 2014). Research would benefit from focusing on the most stigmatized groups from an intersectional perspective (Yoshida, 2013).

## Intersectional reflexivity

Intersectionality-informed mental health, which extends the notion of trauma-informed assessments, recognizes that the emotional pain and distress of oppression can lead to adverse mental health outcomes. In such a context, nuanced emotional trauma histories contribute valuable information to diagnostic decisions and treatment plans (McGibbon, 2012; Shimmin et al., 2017). Engaging in intersectional reflexivity creates trustful, safe therapeutic spaces for socially excluded clients, especially for those who experience multiple layers of social identities and systems of oppression in their lives (Belkin & White, 2020). The critical methodology of intersectional reflexivity is a process of self-understanding (Jones and Calafell, 2012). It comprises explorations of body, affect, and performance to facilitate possibilities of intercultural connections and solidarity (Eguchi & Baig, 2018).

However, intersectional reflexivity can be complicated and often an uncomfortable process even for the most critically minded therapists, as Watt (2007) defines in the Privileged Identity Model. The unraveling of layers of *white* privilege can involve emotional pain for *white* people (Evans-Winters & Hines, 2020; Ringrose, 2007; Todd et al., 2010). *White* Fragility, a term coined in 2011, posits that for many reasons (mostly centered around protecting their self-concept and self-worth), it can sometimes be difficult for *white* people to appreciate the impact of continental ancestry and migration background in reflections on social identity (DiAngelo, 2011, 2018). One of the most significant points of intersectionality theory is that privilege is at any given time tied to many local actualities and embodied experiences (Crenshaw, 2019).

Epistemologically, as a model of bridging rather than breaking, intersectionality is a way of recognizing plurality in the analysis of social inequalities whilst thinking in global terms involving diverse societies and communities in order to elevate a more fully inclusive “we” in the politics of belonging (Yuval-Davis, 2011). This does not exclude *white* women, nor men, from the field of intersectionality. However, it is important to stay rooted in the critical epistemology of Black feminist thought from within which intersectionality emerged, even in contemporary theoretical advancements (Collins & Bilge, 2016). Acknowledging racialization and racism is central to intersectional critiques of power and privilege (Collins, 1989; 2000; Combahee River Collective, 1977/1983; Davis, 1983; hooks, 1981; Lorde, 1984). In mental health science, intersectional reflexivity means that the impact of culturally-mediated, ideology-related information should be fundamental to diagnosis and definitions of what constitutes health and sickness, so the categories used must be culture-specific, or as close to it as possible (Fernando & Moodley, 2018). Furthermore, a critique of Western mental health science's occupational culture should be included in the curriculum (Fernando, 2017; Lazaridou & Heinz, 2021).

## What's next? On solidarity and further decolonial developments

The anxiety-fueled stance underpinning many people's engagement in the ideology of colorblind egalitarianism is a belief that racism arises from acknowledging differences between the socially-mediated cultural codes of various racialized groups (Fryberg & Stephens, 2010). By encouraging the notions of homogeneity and monoculturalism, this stance devalues and denies the reality of cultural diversity in society. Spivak’s (1988) strategic essentialism, by stark comparison, is an avenue through which activists and scholars can safely interrogate the relationship between power and privilege and consider the political implications of “knowledge” to incite equitable structural transformations. Historically, in the allied fields of psychiatry and clinical psychology, substantial differences between African heritage groups were neglected to wrongly justify social hierarchies as supposedly “natural” consequences of imaginary evolutionary narratives favoring *whiteness* (Heinz, 1998).

Derogatory stereotypes attached to People of African Heritage may well have come about from how *white* colonial rulers viewed People of African Heritage (Olusoga, 2015). Strategic essentialism builds on these pre-existing minimizations, drawing the focus on community building to facilitate economic growth, well-being, health, and empowerment. People of African Heritage are doing this for People of African Heritage (Adi, 2018). At the global nexus of knowledge and power, dominant mainstream discourses about racialized communities (whether that be People of African Heritage, Jewish people, People of Color, or asylum seekers and refugees) tend to dictate who deserves to speak and whose perspectives are given credence as intelligent and valid (Aina, 2010).

Historically, intelligence conceptualizations and differential educational attainment became conflated, and the reported statistical variance is between 51% and 75% (Rohde & Thompson, 2007). However, in determining those variations, the impact of *white*-Eurocentric bias in the formal and hidden curriculum, as well as massive income disparities, were largely ignored. Diversification efforts may require a particular focus on prevention and early intervention. However, mental illness stigma creates a culture of “silence”, inhibiting people from actively choosing appropriate and prompt interventions and support (Wynaden et al., 2014). Additionally, the widespread damaging psychological effects of biased projections on racialized students’ performance capacities also needs to be acknowledged. Stereotypes and assumptions are an integral part of the matrix of socially constructed boundaries that cut across circles of human concern in Eurocentric Western education (Lebakeng et al., 2006). These stereotypes and boundaries between people (e.g., insider/outsider, native/migrant, *white*/Other) are codified into Western systems’ structures (policies and practices) (Quijano, 2000).

In turn, these stereotypes and assumptions place obstacles in the paths of racialized individuals and groups, limiting their access to resources (such as project funding) that places them and their Othered “knowledge” outside of the circle of human concern (Barongo-Muweke, 2016). The deliberate inclusion of non-*white* minority bodies, minds, and discourses—on the other hand—in higher education programs and curriculum is a precious chance for students to gain from a conscious, deliberate push toward the habit of questioning the coloniality of the global knowledge–power nexus (Kindiki et al., 2019). Decolonizing training in culture and migration mental health is (a) a long-term commitment to rethinking racism as a combination of racialized prejudice plus social and institutional power (military power, international political power, agricultural power, technological power, domestic socio-political power, and so on) (Bidol, 1972; Barndt, 1991; Sivanandan, 1990), and (b) a position that challenges the numerous disturbing attitudes and behaviors ingrained in *white* mental health facilities that undermine the legitimacy of immigrant and racialized patients’ lived experiences. What is advocated here goes far beyond a request that *white* mental health services be friendlier to immigrant and racialized patients; it is more about raising awareness of the structural level racism in mental health care facilities, and the negative repercussions for racialized patients being exposed to harm, injustice, and racist abuse in those settings (Fernando, 2017). Decolonizing higher education institutions can help students gain critical awareness about intercultural knowledge's multiplicity (Ramrathan, 2016), thereby helping to prevent the formation of biases as part of counteracting racism and many other forms of discrimination (Odora Hoppers & Richards, 2012).

It is not the responsibility of those at the bottom of racism-based inequality structures, outside of the circle of human concern, to create a fairer society. The onus must be upon the mindset of those in power to level the playing field (Thompson et al., 2003). Harnessing the potential of political solidarity with privileged people in positions of influence accelerates progress (Kindiki et al., 2019). For proponents of colorblind egalitarianism situated within the circle of human concern, this means gaining a critical awareness of how Western institutions are an essential contribution to the transmission of social heritage (including attitudes and stereotypes) and thereby reflect a mixture of theories, ideologies and socio-political relations historically embedded in *whiteness*.

## Expanding professional competence training in culture and migration mental health

Researchers have offered many ways that the fabric of training could be refashioned to expand professional competency in culture and migration mental health (Motulsky et al., 2014). For instance, epistemic psychopolitical validity is a powerful epistemological approach to moving beyond the hindrances of dominant *white* discourses (Prilleltensky et al., 2008).

Within a framework focused on developing an integrated social justice concept in students, the reading lists regularly step outside of traditional *whiteness* to create more healthy versions by incorporating marginalized literature (Goodman et al., 2014). Such an approach encourages students’ critical consciousness and enriches their approach to racism affected individuals in clinical practice (Ainslie, 2013; Comas-Diaz, 2007; Sue et al., 2007). For example, the Clinical Doctorate Program at the UK's University of East London introduced a series of workshops: Decolonising *White* Psychology (for third-year students), Talking *Whiteness* (for second-year students) and Introduction to *Whiteness* (for first-year students) (Wood & Patel, 2017).

The content reflects a commitment towards fostering critical consciousness in students by encouraging them to learn about non-Western, marginalized psychological orientations and healing practices (Nwoye, 2015; Rochford, 2004). A theoretical application of inclusion-centered intersectionality tells us that various forms of discrimination in addition to the usual psychological demands of academia may provoke more severe burnout, thus precipitating greater levels of depression (Choo & Ferree, 2010). The inclusion of Black People and People of Color in academic institutions is essential at each level of skilled labor and each student population level. For racialized students, the phenomenology of institutional *whiteness*, both experientially and epistemologically—that is, the lack of lecturers and therapists who look like them—is disillusioning (Lazaridou & Heinz, 2021). Inclusion, in this sense, produces higher social mobility levels and innovation and can contribute to an organizational culture oriented towards more equitable distributions of research grants and a more robust production of equitable knowledge (Vught, 2009). Instead of denying the reality of “experts-on-their-own-experiences”, proponents of colorblind egalitarianism should view intercultural encounters as opportunities to listen deeply and learn (Clark & O’Donnell, 1999).

Institutional changes are necessary for a mental health system that serves all patients fairly and equitably, irrespective of continental ancestry or migration background or skin color. The onus is on every mental health professional and every educator of mental health professionals to hold the institutions they work in accountable. All of which forms a part of socialization (through education) where students can learn to value the cultural diversity of their educators, as well as the cultural diversity of all humanity (Kindiki et al., 2019). When racialized students enter *white* spaces of higher education, they add layers to their vulnerability. What they deserve in return is to be met by people who are continuously engaging in radical and critical education outside of the ethnocentric norm with humility (Gonçalves, 2011). Deconstructing institutional racism requires us to transcend a limited focus on individual perpetrators and towards the inequities racism produces. It is necessary for committed social critics to not only trace racialized/“ethnic” inequities back to the structures and systems of power that perpetuate them, but it is also crucial to present ideas for moving toward future equitable transformations (hooks, 1989a, 1989b). Instead of an essentialization of differences approach or promotion of universal, equal rights binary approaches to social change, understanding, counteracting, and finally eradicating racism in all its forms is fundamental to fully realizing the ambition of equality.
